# Long-term antibody dynamics challenge the paradigm of lifelong homotypic immunity to dengue virus

**DOI:** 10.1073/pnas.2606206123

**Published:** 2026-05-27

**Authors:** Jair Andrade, Adrien Mitard de Girardier, Angkana T. Huang, Darunee Buddhari, Marco Hamins-Puertolas, Maria-Theresa Alera, Mary Noreen Chua, Taweewun Hunsawong, Derek A. T. Cummings, Stephen Thomas, Heather Friberg, Jeffrey R. Currier, Adam Waickman, Aaron Farmer, In-Kyu Yoon, Kathryn Anderson, Alan L. Rothman, Henrik Salje

**Affiliations:** ^a^ Department of Genetics, University of Cambridge, Cambridge CB23EH, United Kingdom; ^b^ Department of Virology, Armed Forces Research Institute of Medical Sciences, Bangkok 10400, Thailand; ^c^ Microbiology and Immunology, State University of New York Upstate Medical University, Syracuse, NY 13210; ^d^ School of Medicine, University of California, San Francisco, CA 94143; ^e^Chong Hua Hospital, Cebu City 6000, Philippines; ^f^Department of Epidemiology, Johns Hopkins Bloomberg School of Public Health, Baltimore, MD 21205; ^g^ Viral Diseases Branch, Walter Reed Army Institute of Research, Silver Spring, MD 20910; ^h^ International Vaccine Institute, Seoul 08826, South Korea; ^i^ Institute for Immunology and Informatics, Department of Cell and Molecular Biology, University of Rhode Island, Providence, RI 02903

**Keywords:** dengue, immunity, antibody kinetics, epidemiology, force of infection

## Abstract

Immunity following infection with the four dengue virus serotypes (DENV1-4) remains difficult to define. Reports of individuals being reinfected with the same serotype challenge the paradigm that infection induces lifelong homotypic immunity. However, the frequency of these events and their importance for shaping immune profiles remain unknown. Here, we used data from three cohorts (N = 4,268 total participants) in two highly endemic settings (Cebu, Philippines, and Kamphaeng Phet, Thailand), which included long-term follow-up of individuals (mean follow-up of 8.8, 1.9, and 5.0 y). These data allowed us to elucidate age-specific patterns of infection and immunity, and to quantify individual long-term antibody titer dynamics following infection. We formulated mathematical models to explain these patterns, allowing for the possibility of progressive loss of immunity to homotypic reinfection. At the individual level, we found that, in the absence of subsequent infection, antibody titers exhibit a steady long-term decay following incident infections (half-life of 7 to 8 y), with the rate of decay slowing with increasing age. At the population level, incorporating homotypic reinfection was required to explain the age-specific dynamics of infection and immunity observed in our cohorts. We estimated that in highly endemic settings such as the Philippines, 60% of individuals have been homotypically reinfected by the age of 40 y. Our findings highlight homotypic reinfections as a key feature of endemic DENV settings and suggest that vaccines mimicking natural infection might not be expected to provide lifelong protection against infection.

## Significance

The four serotypes of dengue virus (DENV1-4) pose a major threat to public health worldwide. The paradigm that individuals can be infected only once per lifetime by each serotype has been challenged by reports of homotypic reinfections; however, the frequency of these events and their importance for shaping immune profiles remain unknown. Using mathematical models fitted to measurements of neutralizing antibody titers from individuals in long-term cohorts living in highly endemic regions, we provide comprehensive evidence that widespread homotypic reinfections are required to explain population-level patterns of infection and immunity over time and across age groups. Our findings have important implications for understanding the role of immunity in driving dengue ecology and for vaccine development and use.

The four serotypes of dengue virus (DENV1-4) are transmitted by *Aedes* mosquitoes and cause >100 million symptomatic infections in humans each year (1). Although vaccines should form the foundation of a comprehensive countermeasure strategy, complex and poorly understood patterns of immunity following infection and vaccination have hampered the development of efficacious dengue vaccines (2). The prevailing paradigm holds that infection with one serotype confers long-lasting homotypic protection and short-term heterotypic protection, such that individuals experience only one infection per serotype over their lifetime. Furthermore, strong evidence indicates that secondary heterotypic infections increase the risk of severe disease (3). Recent studies have challenged this paradigm by suggesting the possibility of homotypic reinfection (repeated infection by the same serotype) (4, 5) and continuous boosting of antibody titers through repeated exposure in endemic settings (6, 7). However, this evidence is largely indirect, deriving from models applied to case data or from relatively short longitudinal cohorts (<5 y). Such studies are insufficient to capture multiple sequential infection events or are conducted in settings without endemic circulation of all serotypes, thereby limiting opportunities for homotypic re-exposure (6, 7).

Reconstructing infection histories, even in carefully followed cohorts in endemic settings, remains a challenge as disease surveillance identifies only a small fraction of total cases (8). It is through the analysis of changes in antibody titers that we can obtain a realistic picture of the true incidence of DENV infection (9). However, even this picture remains incomplete given that many cohort participants will have experienced infections prior to enrollment. The task is further complicated by cross-reactivity among DENV serotypes and with other flaviviruses [e.g., Zika virus (ZIKV)] as well as by imprinting, where across an individual’s history of infections, antibody responses remain biased toward the viral serotype encountered during the first infection, a phenomenon similar to the original antigenic sin observed in influenza (10, 11, 12).

At the population level, age-stratified antibody titer patterns should reflect long-term transmission dynamics. Research on immune dynamics has established that DENV exposure induces a rapid increase in antibody titers, followed by substantial decay within the year following infection (9, 13). However, it is still unclear to what extent titers continue to decay over the long term, or whether subsequent exposures are required to maintain elevated titers.

To address these knowledge gaps, we used data from three cohorts with regular blood draws and active disease surveillance from two highly dengue-endemic regions where all four serotypes cocirculate (14). Two cohorts are from Cebu in the Philippines: The Immunological Correlates of Clinical Outcomes in a Tetravalent Dengue Vaccine Cohort (NMC) and The Prospective Cohort Study of Influenza and Dengue Infection in Children and Adults, Cebu City, Philippines (CPC). The third cohort is the Kamphaeng Phet Family Cohort Study (KFCS) from Kamphaeng Phet, Thailand. We analyzed antibody titer trajectories in participants to characterize the long-term dynamics of infection and immunity. Based on these observations, we formulated mathematical models to evaluate the hypothesis that homotypic reinfection is a key feature of endemic DENV transmission.

## Results

### Long-Term Patterns of Infection.

**Fig. 1.**
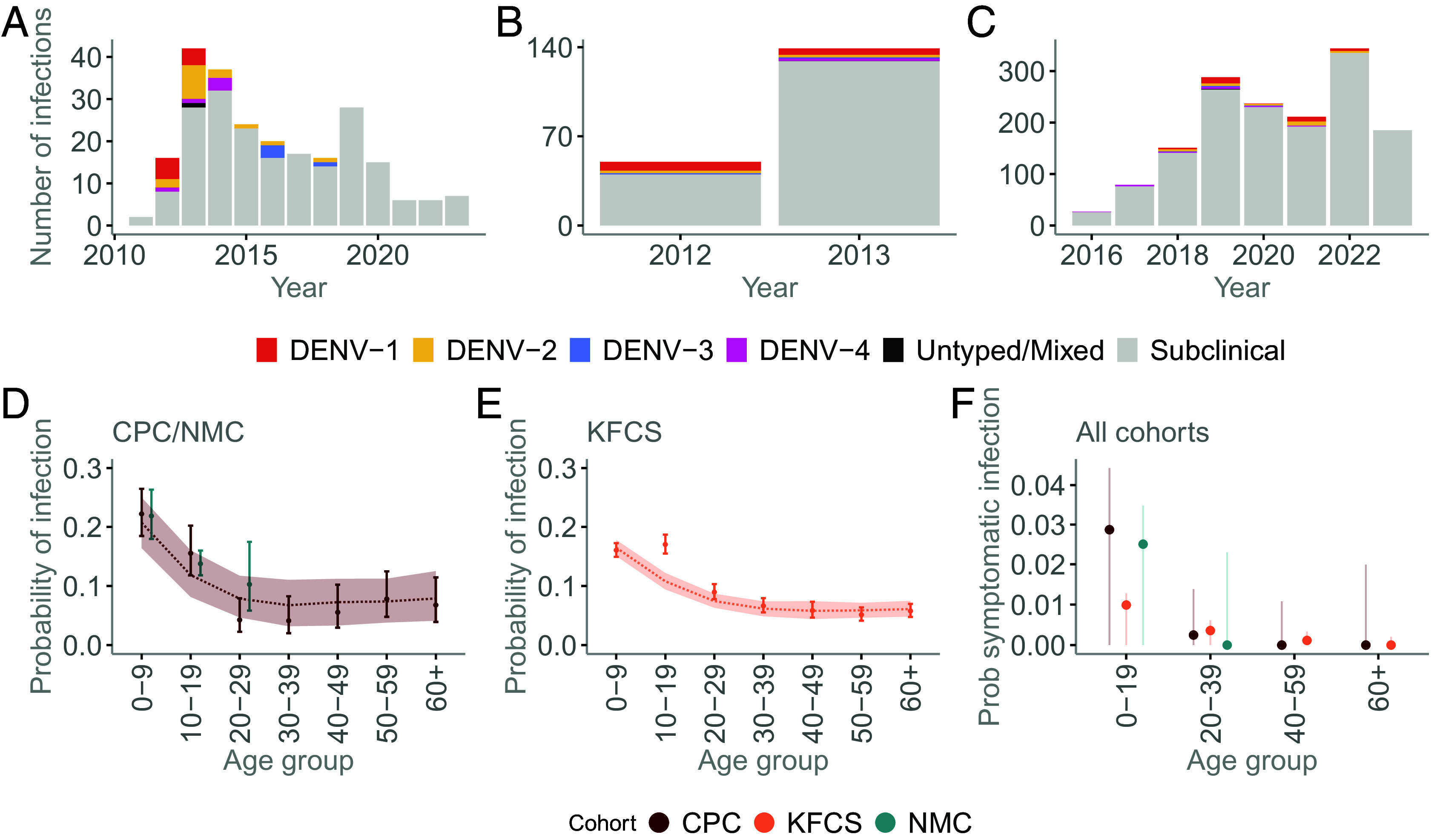
Infections in the cohorts. (*A*–*C*) Annual number of detected dengue virus (DENV) infections in the (*A*) NMC, (*B*) CPC, and (*C*) KFCS cohorts. (*D*) Age-specific probability of DENV infection in the CPC and NMC cohorts. Points show observed infection probabilities, calculated as the proportion of individuals infected among those with blood samples collected in each age group; error bars denote 95% binomial confidence intervals (Wilson method). Shaded ribbons correspond to the 95% uncertainty interval of the infection probability predicted by the catalytic model fitted to the CPC data. The dashed line denotes the median model prediction. CPC is shown in dark red and NMC in teal. (*E*) Same as panel *D*, but for the KFCS cohort. (*F*) Age-specific probability of symptomatic dengue virus infection by cohort. Points show the proportion of blood draws with symptomatic infection in each age group, with vertical lines indicating 95% binomial confidence intervals (Wilson method). CPC is shown in dark red, KFCS in light orange, and NMC in teal.

Our data come from three cohorts. The NMC study comprised individuals who received a placebo as part of a phase III vaccine trial and were followed with annual blood draws and additional sampling during illness events for up to 11 y (1,872 total blood draws from 194 individuals, median age at recruitment of 9 y, range 2 to 14). Of the participants, 13 were seronegative at enrollment, 159 were seropositive, and 22 had unknown baseline serostatus as they had a confirmed infection prior to their first regular blood draw. Over 1,710 person-y of febrile illness surveillance, there were 34 PCR confirmed symptomatic DENV infections (9 DENV1, 15 DENV2, 4 DENV3, 5 DENV4, and 1 unknown serotype), resulting in a mean incidence of 2.0 symptomatic infections per 100 person-y (Fig. 1*A*).

To identify subclinical infections, we used a cut-point of a 1.18 increase in the mean log_2_ PRNT titers across the four serotypes between sequential annual blood draws. This threshold was informed by a previous analysis on this dataset that used full titer trajectories to identify infections, and gives a sensitivity of 95%, a specificity of 99% and identifies 97% of the confirmed PCR infections (*SI Appendix*, Figs. S1 and S2) (15). Using this definition, we identified an additional 202 subclinical infections, resulting in a mean incidence of 11.8 subclinical infections per 100 person-y, and a total incidence of 13.8 infections per 100 person-y. Compared with the previous study (15), which used overall titer dynamics to probabilistically recover subclinical infections (rather than a single cut-point), we estimated a similar number of infections over an equivalent period (160 compared with a mean of 164 across 100 stochastic simulations).

The CPC cohort included individuals (N = 854) of ages 0 to 82 y that ran from 2012–2014. There were 20 symptomatic infections (12 DENV1, 4 DENV2, 2 DENV3, 2 DENV4) over 2 y of follow-up. In addition, we identified 169 additional subclinical infections using a hemagglutination inhibition (HI) 1.6-fold cutoff point as previously identified as optimal (9), implying a mean annual incidence of 11.8 (1.2 symptomatic infections and 10.5 subclinical) infections per 100 person-y.

The Thailand cohort, KFCS, corresponds to a long-term study of a broad-age group (age range: 0 to 93 y) that includes 3,220 individuals (91% seropositive at baseline) who were followed for up to 7 y. We identified 73 symptomatic infections (30 DENV1, 24 DENV2, 4 DENV3, 14 DENV4, 1 mixed serotype). We used a previously published machine learning model tailored to this cohort and fitted to the annual HI titers to identify an additional 1,449 subclinical infections, implying a mean annual incidence of 9.6 (0.5 symptomatic infections and 9 subclinical infections per 100 person-y) (16). We note that a simple cut-off model, as applied to the Philippines cohort, would have yielded a similar incidence (10.5).

Across the three cohorts, we found a consistent decreasing probability of infection with increasing age (Fig. 1 *D* and *E*). Individuals aged 10 to 19 y had, on average, 0.7× the infection risk of those younger than 10 y, decreasing to 0.3 among those aged 20 y or older. In Thailand (Fig. 1*E*), the infection risk among those aged 20 y or older decreased to 0.4 compared with the youngest cohort. In both countries, the probability of infection remained greater than zero across all age groups, including individuals older than 60 y [CPC 0.07 (95% CI: 0.04 to 0.11); KFCS 0.06 (95% CI: 0.05 to 0.07)].

On average, 94% of infections were subclinical (range 86 to 96% across the three cohorts), consistent with prior estimates (9). However, the probability of symptomatic infection differed by age (Fig. 1*F*), decreasing from 0.021 in individuals younger than 20 y (range 0.010 to 0.029 across the three cohorts) to 0.002 in those aged 20 to 59 y in the broad-age cohorts (CPC and KFCS). We observed no symptomatic infections in individuals older than 60 y, despite 112 infections in this age group.

We built a catalytic model to explain the observed patterns of infection by age in two cohorts with a wide range of ages (KFCS and CPC). We considered two competing models that either did or did not allow for individuals to be reinfected by each serotype. The model has three parameters: the average annual force of infection by serotype (allowing for different values in Thailand and the Philippines) and the annual rate of loss of homotypic immunity following infection (shared across both settings, and forced to equal zero in the simpler model). We only allowed for a single infection per year. We fitted these models using a binomial likelihood to the observed age-specific infection counts in 1-y age bands (out of the number of individuals in each band) across the two cohorts (CPC and KFCS).

Our model reproduced the observed age-specific infection patterns (Fig. 1*D*) with per-serotype forces of infection estimated at 0.085 (95% CI: 0.073 to 0.099) in the Philippines cohort and 0.057 (95% CI: 0.055 to 0.058) in the Thai cohort. We estimated that following initial complete protection, there was a decline in homotypic protection of 0.0075 per year (95% CI: 0.0066 to 0.0082), implying that at 1 y following an infection, individuals had 0.0075× the hazard of homotypic reinfection as compared to individuals with no history of infection by that serotype, rising to a relative hazard of 0.075 after 10 y. The model with no homotypic reinfection was significantly less well supported by the data (ΔAIC = 485).

### Individual-Level Titer Dynamics.

**Fig. 2.**
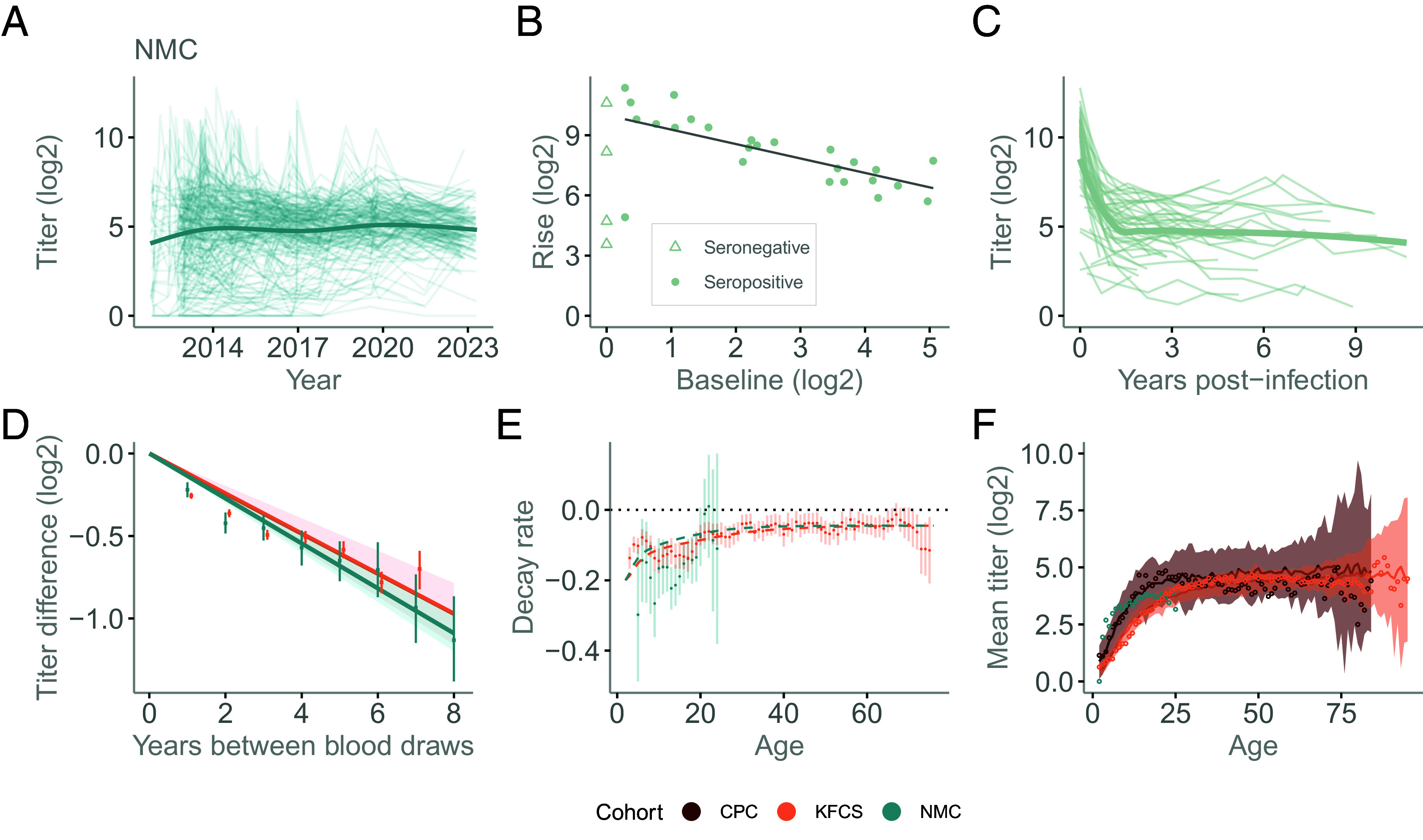
Titer behavior. (*A*) Individual titer trajectories in the NMC cohort. The bold line represents a smoothed trend estimated across individuals. (*B*) Baseline antibody titer vs. titer rise. Points represent observations from individuals with PCR-confirmed infections, with shape indicating serostatus. The solid gray line denotes the linear fit for individuals with seropositive status. (*C*) Individual titer trajectories following PCR-confirmed infection. Thin lines show individual trajectories, and the bold line represents a smoothed trend (LOESS) estimated across individuals. Blood draws associated with infections detected after the PCR-confirmed infection were excluded. (*D*) Change in antibody titers as a function of time between blood draws in the long-term cohorts: KFCS (light orange) and NMC (teal). Points show mean titer differences, with error bars indicating 95% uncertainty intervals estimated via bootstrapping. Lines represent cohort-specific linear fits. (*E*) Age-specific antibody decay rates in long-term cohorts. Points represent 3-y moving-average estimates of decay rates by age, with error bars indicating 95% confidence intervals. Dashed lines indicate estimated age-dependent decay rates, assuming decay decreases with successive infections until the fourth and then stabilizes. (*F*) Age-specific mean antibody titers. Open circles indicate the observed mean titer by age, solid lines show the median predicted by the simulation model, and shaded ribbons indicate the 2.5th to 97.5th percentile interval. Colors indicate cohorts: CPC (dark red), KFCS (light orange), and NMC (teal). Unless otherwise stated, panels consider all individuals, regardless of baseline serostatus.

We next evaluated the evolution of individual titers in the long-term cohorts. In the Philippines (NMC), baseline seropositive participants exhibited stable mean log_2_-PRNT titers across the four serotypes, rising modestly from 4.7 (95% CI: 4.4 to 5.1) at enrollment to 4.9 (95% CI: 4.7 to 5.1) 10 y later (Fig. 2*A*). Similarly, in Thailand (KFCS), mean log_2_-HI titers among baseline seropositive individuals increased slightly from 3.8 (95% CI: 3.6 to 4.0) to 4.1 (95% CI: 3.9 to 4.3) over 7 y. HI and PRNT titers were strongly correlated (ρ = 0.89; *SI Appendix*, Fig. S3) in a subset of individuals with measurements from both assays.

We next characterized the rise in titers following infection, focusing on PCR-confirmed symptomatic cases with known infection dates, to obtain a clearer understanding of individual-level titer dynamics. In the Philippines, we found that log_2_-PRNT titers were, on average, 8.0 (95% CI: 7.2 to 8.7) higher in acute illness samples than prior to infection (*SI Appendix*, Fig. S4). However, the rise was highly dependent on the preinfection titer, with the magnitude of the rise being 0.7 lower for each log_2_-PRNT titer increase in preexisting titer in seropositive individuals (Fig. 2*B*). Seropositive individuals with preinfection log_2_-PRNT titers of <3 had a mean rise of 9.1 (95%CI: 8.2 to 10.0), whereas individuals with a log_2_-PRNT titer of >3, had a mean rise of 6.9 (95%CI: 6.4 to 7.5). A similar pattern was observed in mean log_2_-HI titers around symptomatic infections in Thailand, with a mean rise of 6.8 (95%CI: 6.2 to 7.4), ranging from 7.3 (95%CI: 6.6 to 8.0) in those with preexisting titers of <2 (approximately 3 in the log_2_ PRNT scale), to 5.7 (95%CI: 4.7 to 6.8) with preexisting log_2_ HI titers of >2.

The extended duration of follow-up in NMC and KFCS enabled us to examine the short- and long-term titer dynamics following each PCR-confirmed infection. We found that individual titers in both cohorts exhibited biexponential decay (Fig. 2*C*). We fitted separate exponential decay models: one to blood draws within 1 y of infection, and another to blood draws more than 1 y after infection. We excluded person-time following subsequent infection events (PCR confirmed and subclinical) to remove the influence of boosts from new infections. In NMC, we estimated that in the first year following infection, there was an average decay in log_2_-PRNT titers of 5.2 per year (95%CI: 4.4 to 6.1, corresponding to a half-life of approximately 2 mo). Beyond this time, there was an average continual slow decay of 0.15 per year (95%CI: 0.08 to 0.21), corresponding to a half-life of 6.7 y (95%CI: 4.8 to 12.5 y). In Thailand, we estimated an initial average decay in log_2_-HI titers of 5.7 per year (95%CI: 4.8 to 6.5, corresponding to a half-life of approximately 2 mo) and an average continual slow decay in log_2_-HI titers of 0.25 per year [95%CI: 0.15 to 0.34, corresponding to a half-life of 4.0 y (95%CI: 2.9 to 6.7 y)].

As an alternative estimate of the long-term rate of titer decay, we considered the difference in log_2_-titers between all blood draws of each individual, excluding pairs of blood draws with an incident infection between the blood draws (subclinical or symptomatic) as well as samples collected within 1 y of infection to remove the effects of short-term decays (Fig. 2*D*). In the Philippines, we found an average decay rate of 0.14 per year (95%CI: 0.12 to 0.15), consistent with the value estimated from PCR-confirmed infection events, with similar estimates across the four serotypes (range: 0.09 to 0.18 per y) (*SI Appendix*, Fig. S5). We conducted a sensitivity analysis in which we evaluated higher and lower thresholds for defining subclinical infections with consistent results (*SI Appendix*, Fig. S6). A similar decay pattern was observed in the Thai dataset, with a mean decay rate of 0.12 per year (Fig. 2*D*). Results were consistent across different imputation values below the limit of detection (*SI Appendix*, Fig. S7).

We found that the long-term titer decay rate varied by age group, with estimated mean decay rates of 0.25 (95% CI: 0.14 to 0.37) and 0.14 (95% CI: 0.11 to 0.16) per year among individuals <10 y in the NMC and KFCS cohorts, respectively, decreasing to approximately 0.03 (95% CI: −0.13 to 0.18) and 0.06 (95% CI: 0.04 to 0.08) per year among individuals aged 20 to 30 y (Fig. 2*E*). As younger individuals are more likely to experience primary infections, these findings are consistent with more rapid decay in titers following primary infections compared with subsequent infections (9). Notably, in Thailand, the decay rate stabilized among individuals >40 y at a level that remained significantly different from 0 (mean 0.06; 95% CI: 0.04 to 0.07).

### Population-Level Titer Dynamics.

At the population level, we found substantial differences in mean log_2_-titers by age (Fig. 2*F*). In the CPC Philippines cohort, mean titers increased by 0.20 log_2_-HI titers per year over the first 20 y of life (0.21 log_2_-PRNT titers per year in the NMC cohort). Titers peaked at 22 y of age (4.8 log_2_-HI titers, 95% CI: 4.4 to 5.3). After this age, mean log_2_-HI titers remained largely stable, exhibiting only a small but statistically detectable decline of 0.008 log_2_-HI titers per year (95%CI: 0.004 to 0.013; *P*-value for no decline of <0.001). Similarly, in Thailand, mean titers increased by 0.16 log_2_-HI titers per year over the first 20 y of life, but peaked later at 48 y of age, followed by a small but statistically significant decline of 0.008 log_2_-HI titers per year (95%CI: 0.004 to 0.011; *P*-value < 0.001).

The rates of long-term titer decay we found in individual-level data (0.15 to 0.25 log_2_-HI titers per year) were around 20 to 30× greater than the observed reduction in titers by age in older ages (0.008 log_2_-HI titers per year). We evaluated whether homotypic reinfection could account for these differences. To do this, we expanded the catalytic infection model to incorporate titer dynamics by mapping each simulated infection event to a titer trajectory. These trajectories were generated using an exponential decay function to represent long-term antibody decline. We restricted ourselves to representing the long-term dynamics, given that blood draws were obtained primarily at yearly intervals, thereby limiting our ability to infer short-term dynamics. Decay rates and starting titers varied by infection sequence. The former were specified according to patterns observed in the NMC and KFCS cohorts (Fig. 2*E*), whereas the latter were estimated. We then aggregated individual trajectories by age, yielding a population-level average. We fitted the model to age-specific infection and population titer data from CPC and KFCS cohorts and recovered the observed mean titers per age (Fig. 2*F*). The resulting estimates of the force of infection (0.085 in the Philippines and 0.054 in Thailand) and the annual loss of homotypic immunity (0.0064 per year) were very similar to those estimated in our original model using individual infection incidence data only (*SI Appendix*, Table S1).

**Fig. 3.**
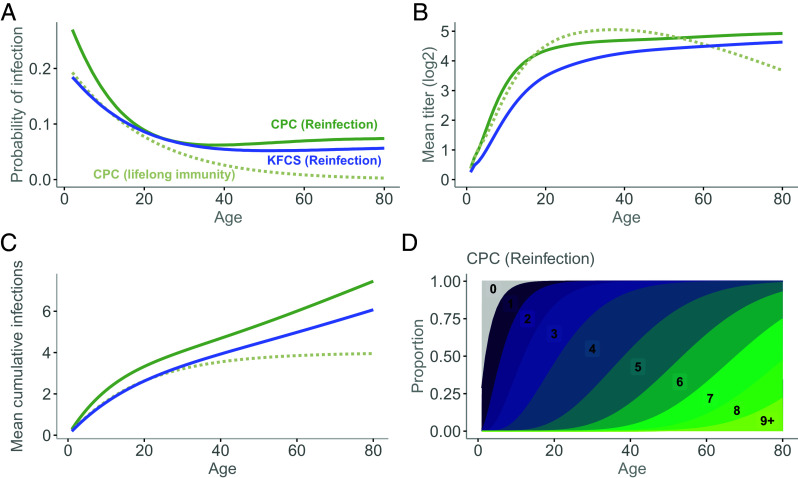
Expected population patterns of infection and immunity under competing scenarios. Results are based on simulations of infection histories for 10^7 individuals and their corresponding titer dynamics, in which parameter values are set to the maximum likelihood estimates obtained by fitting models that account for infection and antibody dynamics to the CPC and KFCS cohorts. In Fig. 3 *A*–*C*, solid lines indicate simulations based on parameter estimates obtained under the assumption of homotypic reinfection, whereas dotted lines indicate simulations based on parameter estimates obtained under the assumption of lifelong immunity to homotypic reinfection (i.e., annual loss of immunity set to zero). Results based on parameter estimates fitted to the KFCS cohort under the lifelong homotypic immunity assumption are omitted for clarity, as they are qualitatively similar to those based on the CPC cohort and do not provide additional insight. (*A*) Age-specific probability of infection by scenario. (*B*) Age-specific mean antibody titers by scenario. (*C*) Age-specific mean cumulative number of infections by scenario. (*D*) Age-specific distribution of the cumulative number of infections for a simulated population under the homotypic reinfection scenario, using parameter estimates from the CPC cohort. Labels within the plot denote the cumulative number of infections.

We found that in competing models that did not allow for homotypic infection, population titers sharply decay at older ages, a pattern incompatible with our observations (*SI Appendix*, Table S2 and Fig. 3 *A*–*C*). In addition, models that allowed for declining or increasing force of infection, or substantial heterogeneity in infection risk, could not recover the observed age-specific patterns of infection and antibody titers (*SI Appendix*, Figs. S8 and S9).

Finally, we used our fitted model to estimate the number of homotypic reinfections by age. We estimated that in a setting such as the Philippines, 14% of individuals experience homotypic reinfection by age 20 y, increasing to 91% by age 60 y (Fig. 3*D*). On average, individuals are expected to have experienced 3.3 infections by age 20 y and 6.0 by age 60 y. In a context such as Thailand, where the force of infection is lower, individuals are expected to experience an average of 2.6 infections by age 20 y, increasing to 5.0 by age 60 y.

## Discussion

Using data from three cohort studies in highly endemic settings with follow-up of individuals across a wide age range and for long time-periods, we have been able to track the long-term patterns of infection and dynamics of antibody titers, allowing us to critically assess competing hypotheses of homotypic resusceptibility. We have shown that among seropositive individuals, in the absence of reinfection, there is a steady long-term decline in antibodies. Further, the short-term rise in antibodies following infection also gets progressively smaller as individuals obtain higher baseline titers. Nevertheless, while the risk of infection declines with increasing age, we identify clear seroconversions in individuals of all ages. We could only recover this observation in models that allow for homotypic reinfection.

The drivers of homotypic reinfection remain unclear, but may include a combination of waning antibody titers leading to resusceptibility and the emergence of antigenically distant homotypic viruses (11, 17). Consistent with this latter hypothesis is the finding that viruses in Thailand increased in antigenic distance from each other over time (17). As we could not identify infecting serotypes for the subclinical infections, we could not identify serotype-specific differences, nor be able to disentangle the probability of homotypic infection as a function of infection parity. It may be that the first infection in life remains the most important exposure that dictates future immune responses, and that homotypic reinfection only occurs with nonprimary infecting serotypes, although the lack of a clear dominant serotype-specific response across sequential infections suggests that original antigenic sin does not fully drive lifetime responses (*SI Appendix*, Figs. S10 and S11).

The implications of homotypic reinfection for disease risk remain unclear. We note there were no symptomatic DENV infections in older individuals in our cohorts, suggesting that homotypic reinfection is generally not symptomatic. This is consistent with studies that have explored long-term hospital data to show that repeated dengue hospitalizations were consistently from infection by heterotypic serotypes (18). Similarly, it remains unclear whether homotypic reinfections can contribute to onward viral transmission. As our inferences are based on immune responses only, the homotypic reinfection events may not be infections in the traditional sense, and could be more precisely described as immune-stimulating events. We do know that individuals with preclinical and asymptomatic infection are infectious to mosquitoes (19, 20), however, the extent to which this applies to homotypic reinfections is unknown. Any contribution of homotypic infection to onward transmission is likely to generate lower estimates of R_0_ for a fixed mean force of infection than when sterilizing immunity is assumed.

The implications of our findings for vaccines and other interventions are complex. Any interventions (e.g., vaccines, Wolbachia-infected mosquitoes) or secular trends (e.g., falling birth rates) that reduce the force of infection will limit opportunities for homotypic reinfection (21, 22). In the medium term, this would lead to lower titers in older age groups, exposing these more vulnerable individuals to potentially severe symptomatic illness (23). On the other hand, live vaccines typically seek to mimic the immune responses from natural infection, and our findings suggest that DENV vaccines are therefore unlikely to provide long-term protection from infection, especially if DENV vaccines stimulate lower antibody titers than natural infection. In this latter scenario, the impact on the force of infection from vaccines may be more limited, with titer levels in older individuals largely unaffected (15). The implications for vaccinated individuals in nonendemic populations, such as travelers, remain less clear, although booster doses will probably be required to maintain immunity. Cellular immunity generated by vaccines and natural infection also appears important in disease pathogenesis and may be more long-lived (24).

Our study is subject to limitations. We were not able to make serotype-specific inferences, we therefore cannot identify serotype-specific effects. In addition, Zika virus infection or Japanese encephalitis vaccination in children may lead to increases in titers, however, these would be single events and would not explain the observed patterns of infection over all age groups. Further, measured antibody specific titers will depend on the specific viruses used in the assay and their antigenic relatedness with circulating viruses (11). As the status and dynamics of DENV-specific memory B cells and long-lived plasma cells may differ depending on the number of infections and the specific infecting strain, we allowed the decay rate to vary across successive infections, informing this relationship using the observed change in decay rate with age. However, this assumption does not account for independent age effects irrespective of infection sequence.

This study has identified that homotypic dengue virus reinfection is frequent. Our findings are consistent with a growing realization that homotypic reinfection is more common across pathogens than long believed, including for viruses such as measles, which has long been seen as an example of lifelong sterilizing immunity (25–27). Our inferences were only made possible through detailed and frequent immunity measurements over long time periods combined with mathematical modeling. This level of detail is rarely available for pathogens. The extent to which homotypic reinfection is a phenomenon with limited impact on disease incidence or a critical driver of viral evolution and pathogen ecology now needs exploration.

## Methods

### Cohorts Description.

We used DENV serological data from three longitudinal cohorts. Two cohorts were located in the Cebu region, Philippines, and one from Kamphaeng Phet, Thailand.

### NMC Study.

The NMC study was part of the Dengvaxia phase III trial that followed children and teenagers after vaccination since 2011 (15). In this study, we used data collected up to April 2023 from the placebo group, which consisted of 194 participants (median age at enrollment: 9 y, range: 2 to 14 y). These participants had yearly follow-up visits where blood samples were collected. All visits happened around the same time to have a comparable cumulative yearly force of infection. Some participants who enrolled early in the study had more frequent visits in the first year of follow-up (corresponding to the vaccine doses). Additional blood draws were taken following symptom onset and were used to confirm DENV infection and infecting serotype via reverse-transcriptase polymerase chain reaction (RT-PCR). All blood samples were tested using DENV serotype-specific PRNT assays. PRNT titers were measured on a continuous scale, with a lower limit of detection of 10. Individuals were considered seropositive at enrollment if they had positive PRNT titers (>10) to any serotype in the first (baseline) blood draw. 22 individuals had confirmed infections prior to their enrollment, captured by disease surveillance conducted as part of the vaccine trial, and their baseline serostatus was unknown.

### CPC Study.

The CPC study was shorter in duration but covered a wider age group in the same area (14). We used DENV serotype-specific HI titers from the baseline blood collection. Those were measured on a discretized scale using twofold serial dilutions, with a lower limit of detection of 10. We removed all individuals equal to or younger than 1 y to avoid the influence of residual maternal antibodies. After this restriction, the dataset comprised 851 participants.

### KFCS Study.

KFCS stems from an ongoing cohort study in Kamphaeng Phet, Thailand (16). The study started in September 2015 to identify immunological correlates of protection from DENV infection and illness, as well as factors shaping DENV transmission in multigenerational households. This dataset includes yearly follow-up of participants through February 2023, during which serum samples were obtained, along with active illness and household investigations triggered whenever a participant reported a fever. Yearly serum samples were tested using HAI, and illness and household investigations included RT-PCR and HI testing. As with the CPC cohort, we excluded all individuals aged 1 y or younger, resulting in 3,174 individuals included in the analysis.

### Titer Representation.

PRNT and HI assays were performed for all four DENV serotypes. Results below the limit of detection (<10) were replaced with a value of five and subsequently transformed onto an adjusted log_2_ scale (9) denoted by $lo{g_{2}}^{*}A$ and defined as

$$lo{g_{2}}^{*}A=1+\frac{log(\frac{A}{10})}{log2}.$$

such that measured titers (A) are mapped as follows: <10 = 0, 10 = 1, 20 = 2, 40 = 3, and so on. Given the strong correlation among serotype-specific titers within a given blood draw, we represent each blood draw by the mean of its four adjusted-log_2_ titers. Consequently, a mean of 0 on the adjusted log_2_ scale refers to undetectable titers for all serotypes and serves as the criterion for determining seropositivity.

To allow comparison between PRNT log_2_*-titers and HI log_2_*-titers, we fitted a linear regression model using a subset of 560 paired samples from the NMC cohort (*SI Appendix*, Fig. S3). We estimated a scaling factor of 0.72 (corresponding to the regression slope).

### Definition of Subclinical Infections.

To identify subclinical infections in the NMC cohort, we used the mean log_2_*-titer difference between two consecutive blood draws (9). We identified the optimal cut point using the results of a modeling study based on a subset of data from this cohort that probabilistically reconstructed individual titer trajectories and infection histories in parallel (15). We assumed that model predictions of infection between two sequential annual blood draws with a probability of infection above 0.9 corresponded to true infections, whereas predictions with a probability below 0.1 between sequential blood draws represented true absences of infection. We then used the R package *pROC* to identify a fixed log_2_*-titer difference between any two sequential blood draws that maximized sensitivity and specificity. We found that a cutoff of 1.18 log_2_*-titer units yielded 95% sensitivity and 99% specificity. This cut point identified 97% of all the PCR-confirmed infections in the study.

In the CPC cohort, we identified infections using the log_2_*-titer ratio between two consecutive blood draws with a 1.6-fold cutoff point, which was previously reported to yield 90% sensitivity and 95% specificity for annual samples (9).

In the KFCS cohort, we used a gradient-boosted regression model tailored to this cohort and fitted to annual HI titers (16). This model identifies infections with 93.3% sensitivity and 98.0% specificity. As a sensitivity analysis, we also applied the ratio method used in the CPC cohort.

### Original Antigenic Sin.

The original antigenic sin refers to the phenomenon in which primary immune responses bias immunity to secondary and subsequent dengue virus infections. Within the first few wk following primary dengue virus infection, memory B cells and plasma cells with the highest specificity and/or affinity for the infecting viral serotype preferentially expand. Following subsequent dengue virus infection, antibody responses differ markedly and are characterized by much higher antibody titers and a broader pattern of neutralization across the four dengue virus serotypes. Antibody titers specific to the serotype responsible for the earlier infection increase substantially and often remain higher than those directed against the currently infecting serotype (28).

We therefore evaluated whether the original antigenic sin is a persistent feature of DENV infection history. If it persists across an individual’s infection history, the neutralization titer specific to the serotype of primary infection would remain substantially higher after each subsequent infection, resulting in a single dominant serotype. Conversely, if the original antigenic sin does not shape DENV infection, the neutralization titer following each infection would be highest for the infecting serotype, resulting in a number of dominant serotypes equal to the number of infections. Accordingly, we identified the highest titer among the four measured titers (one for each serotype) in samples from the NMC cohort collected immediately after infection events. To account for the possibility that some serotypes consistently generate higher titers due to their antigenic properties in the assay, we centered each titer around the mean for that serotype; however, the unadjusted results were nearly identical (*SI Appendix*, Fig. S10). We repeated this analysis in the subset of seronegative individuals (*SI Appendix*, Fig. S11).

### General Long-Term Titer Decay.

We investigated long-term decay using pairs of blood samples from the same individual, ensuring that both samples followed the same infection event. Let $\tau_{j}$ denote the time of the j-th infection, and let $A_{t}$ denote the antibody titer measured at time t. A blood-sample pair $\left(A_{t_{1}},A_{t_{2}}\right)$, measured at times $t_{1}$ and $t_{2}$, is said to belong to the infection event $\tau_{j}$ if

$$\tau_{j}\leq t_{1}<t_{2}<\tau_{j+1}.$$

Infection events include PCR-confirmed and subclinical infections. Enrollment and end of individual follow-up dates are also treated as infection events. This assumption allowed us to consider the entire set of blood draws of an individual. Further, we excluded the first data point following infection to avoid the confounding impact of short-term decay in titers.

To limit the impact of measurement uncertainty, we computed the titer ratio between all the possible sample pairs belonging to an infection event (Fig. 2*D*). Under the assumption that the long-term dynamics of titers follow exponential decay, a linear regression on the $lo{g^{*}}_{2}$ scale over time yields the decay rate:

$$A_{t_{j}}=A_{t_{i}}\exp(-\gamma (t_{j}-t_{i})),$$

$$lo{g^{*}}_{2}(A_{t_{j}})-lo{g^{*}}_{2}(A_{t_{i}})=-\frac{\gamma }{log2}(t_{j}-t_{i}),$$

where the term $\frac{\gamma }{log2}$ denotes the decay rate in the $lo{g^{*}}_{2}$ scale, and its reciprocal corresponds to the half-life. To discern the impact of age on titer decay (Fig. 2*E*), we restricted ourselves to pairs with a maximum gap of 2 y so that the decay can be attributed to a specific age with low ambiguity.

### Age-Specific Incidence Proportions.

We fitted a mechanistic model to the age-specific infection patterns observed in the broad-age cohorts (KFCS and CPC). This model represents the number of infections at age a in cohort c *(*$Y_{a}^{c}$) as a binomially distributed variable, where $p_{a}$ denotes the age-specific probability of infection and $N_{a}$ the number of individuals at risk at age a.

$$Y_{a}^{c}∼binomial(p_{a}^{c},N_{a}^{c}).$$

We employed a simulation approach based on a catalytic framework to approximate $p_{a}^{c}$ by simulating individual infection histories in a theoretically large population and recording ages at infection. To simulate infections, we assumed that individuals become fully susceptible to the four DENV serotypes 1 y after birth, once maternal antibody protection has waned. For each individual, we iterated over each year of life and, for each serotype s=1,2,3,4, simulated infection as a binary outcome with probability

$$1-\exp\left[-\left(1-\psi_{a}^{s}\right)\lambda_{s}^{c}\right],$$

where $\lambda_{s}^{c}$ represents the cohort-specific, per-serotype force of infection, $1-\psi_{a}^{s}$ susceptibility to infection at age a for serotype s, and $\psi_{a}^{s}$ immunity to homotypic reinfection. Note that $\psi_{a}^{s}$ varies by individual, but individual-level indices are omitted for clarity. For simplicity, and given the levels of endemicity in both countries, we assume that $\lambda_{s}^{c}=\lambda^{c}$. Moreover, individuals are fully susceptible to the four serotypes ($\psi_{a}^{s}=0$) before experiencing an infection. For each year of life, we draw a Bernoulli sample to determine whether an individual becomes infected. Since infection with a specific serotype confers temporary protection against the remaining three serotypes, we assume that individuals can be infected by at most one serotype in a given year. Consequently, we select the infecting serotype by performing an additional draw, in which the probability that a given serotype is selected is weighted by ${1-\psi }_{a}^{s}$. We set the immunity to the infecting serotype ($s^{*}$) to one, i.e., $\psi_{a}^{s^{*}}=1$, to reflect perfect homotypic protection immediately after infection.

We represented the decline in homotypic immunity at any age as a linear decay given by the expression

$$\psi_{a+1}^{s}=max(\psi_{a}^{s}-\rho ),0),$$

where ρ denotes the yearly rate of immunity decline after infection.

We then combined individual infection histories to estimate the age-specific proportion of infection. The resulting estimator ${\hat{p}}_{a}$ converges to $p_{a}$ as N→∞ (*SI Appendix*, Fig. S12), and enables likelihood evaluation for any parameter set. Because this procedure is stochastic, likelihood values were computed as the average of 10 simulations with fixed seeds to ensure a well-defined likelihood function, so that each parameter set maps to a unique likelihood value, yielding deterministic and unbiased estimates. Specifically, for each evaluated set of parameter values, we performed 10 simulations, computed the likelihood for each, and then averaged the results. Each simulation consisted of $10^{4}$ individuals to balance computational cost and variance in the likelihood estimate. These deterministic likelihood estimates enabled us to use the derivative-free local optimization algorithm NLOPT_LN_SBPLX (Subplex), as implemented in the NLopt library, to obtain the maximum likelihood estimate and construct profile likelihoods for confidence interval estimation.

### Dynamics of Population Antibody Titers Across Age.

We expanded the infection-generator model to link individual and population-level titer dynamics. Specifically, we incorporated a structure that simulates antibody titers at age a ($x_{a}$) given the individual’s infection history ($\tau_{1},\tau_{2},\cdots ,\tau_{m}$), where each $\tau_{j}$ corresponds to the age at the j -th infection and m to the number of infections.

$$x_{a}=\sum_{j=1}^{m}1_{a\leq \tau_{j}<\tau_{j+1}}A_{0}^{j}\exp(-\gamma_{j}(\tau_{j}-a)),$$

This structure comprises m exponential decay trajectories, with each trajectory j spanning the interval from $\tau_{j}$ to $\tau_{j+1}$, delimited by the indicator function $1_{a\leq \tau_{j}<\tau_{j+1}}$. We restricted ourselves to representing the long-term dynamics, given that blood draws were obtained primarily at yearly intervals, thereby limiting our ability to infer short-term dynamics. Upon each infection, antibody titers were assumed to instantaneously peak at $A_{0}^{j}$ and subsequently decay exponentially at rate $\gamma_{j}$.

We assumed that peak titer magnitude increased with successive infections, consistent with our previous studies (15). However, the data indicate saturation, with titer increases inversely proportional to baseline levels. Accordingly, we modeled peak antibody titers as a first-order saturation process, in which successive peaks increase monotonically while incremental gains diminish over time:

$$\log_{2}^{*}A_{0}^{j}=\phi -(\phi -\log_{2}^{*}A_{0}^{1})\exp[-(j-1)],$$

where ϕ represents the asymptotic upper bound approached by peak antibody titers as the number of infections increases. ϕ and $\log_{2}^{*}A_{0}^{1}$ are treated as estimated parameters.

Furthermore, we postulated that $\gamma_{j}$ decreases with successive infections, consistent with evidence that antibody decay rates decline with age. Specifically, we model this decline using an exponential structure,

$$\gamma_{1}exp[-(j-1)\delta )],$$

where δ governs the rate at which decay slows with repeated infections, and $\gamma_{1}$ denotes the decay rate following primary infections. Importantly, because the evidence indicates that decay rates stabilize at older ages, we assumed that the decline in the decay rate ceases after the fourth infection, yielding

$$\gamma_{j}=\max\left[\gamma_{1}\exp\left(-\left(j-1\right)\delta \right),\gamma_{1}\exp\left(-3\delta \right)\right].$$

We set $\gamma_{1}=0.2$ and δ=0.5 to reproduce the observed pattern in the data (Fig. 2*E*).

Finally, we aggregated individual trajectories by age to formulate the second observation component of the likelihood, which accounts for age-specific mean titers in each cohort denoted by ${\bar{T}}_{a}^{c}$:

$$T_{a}^{c}∼N\left(\log_{2}^{*}x_{a}^{c},\frac{\sigma }{\sqrt{N_{a}^{c}}}\right),$$

*Here* ${\bar{x}}_{a}^{c}$ indicates the age-specific simulated mean titer for cohort c, $\frac{\sigma }{\sqrt{N_{a}^{c}}}$ the standard error, with σ denoting the standard deviation and $N_{a}^{c}$ the number of age-specific measurements in cohort c.

In this modeling framework, we assumed that there was not a baseline minimum titer below the limit of assay detection, which could correspond to a naïve B-cell repertoire.

### Alternative Hypotheses for Population-Mean Titer Patterns.

We evaluated whether alternative force of infection (FOI) assumptions could reproduce the observed age-specific pattern in population-mean titers in the absence of homotypic reinfection (*SI Appendix*, Fig. S8). Specifically, we considered three additional scenarios. In the first scenario (Decreasing FOI), we assumed a time-varying FOI that declines from the birth of the oldest individual to the last observed age of the youngest individual. Conversely, in the second scenario (Increasing FOI), we assumed a time-varying FOI that increases over the same period. In these two scenarios, the FOI varies annually with fixed increments or decrements. The average of these time-varying FOIs is approximately equal to the value estimated for the CPC cohort (*SI Appendix*, Table S1). In the final scenario, we assumed a constant population FOI with heterogeneous individual risk ($r_{i}$), where $r_{i}∼Gamma\left(5,5\right)$.

## Supplementary Material

Appendix 01 (PDF)

## Data Availability

Analysis code and anonymized datasets have been deposited in Github (https://github.com/jandraor/DENV_reinfections) (29).

## References

[1] J. D. Stanaway , The global burden of dengue: An analysis from the Global Burden of Disease Study 2013. Lancet Infect. Dis. **16**, 712–723 (2016). 26874619 10.1016/S1473-3099(16)00026-8 PMC5012511

[2] S. J. Thomas, I.-K. Yoon, A review of Dengvaxia®: Development to deployment. Hum. Vaccin. Immunother. **15**, 2295–2314 (2019). 31589551 10.1080/21645515.2019.1658503 PMC6816420

[3] S. B. Halstead, Dengue. Lancet **370**, 1644–1652 (2007). 17993365 10.1016/S0140-6736(07)61687-0

[4] J. J. Waggoner , Homotypic Dengue virus reinfections in Nicaraguan children. J. Infect. Dis. **214**, 986–993 (2016). 26984144 10.1093/infdis/jiw099 PMC5021223

[5] B. M. Forshey, S. T. Stoddard, A. C. Morrison, Dengue viruses and lifelong immunity: Reevaluating the conventional wisdom. J. Infect. Dis. **214**, 979–981 (2016). 26984147 10.1093/infdis/jiw102

[6] L. W. Alexander , Boosting can explain patterns of fluctuations of ratios of inapparent to symptomatic dengue virus infections. Proc. Natl. Acad. Sci. U.S.A. **118**, e2013941118 (2021). 33811138 10.1073/pnas.2013941118 PMC8040803

[7] R. A. Aogo , Effects of boosting and waning in highly exposed populations on dengue epidemic dynamics. Sci. Transl. Med. **15**, eadi1734 (2023). 37967199 10.1126/scitranslmed.adi1734 PMC11001200

[8] G. Ribeiro dos Santos , Individual, household, and community drivers of dengue virus infection risk in kamphaeng phet province, Thailand. J. Infect. Dis. **226**, 1348–1356 (2022). 35512137 10.1093/infdis/jiac177 PMC9574660

[9] H. Salje , Reconstruction of antibody dynamics and infection histories to evaluate dengue risk. Nature **557**, 719–723 (2018). 29795354 10.1038/s41586-018-0157-4 PMC6064976

[10] D. F. Robbiani , Recurrent potent human neutralizing antibodies to Zika virus in Brazil and Mexico. Cell **169**, 597–609.e11 (2017). 28475892 10.1016/j.cell.2017.04.024 PMC5492969

[11] L. Wang , Antigenic distance between primary and secondary dengue infections correlates with disease risk. Sci. Transl. Med. **16**, eadk3259 (2024). 38657027 10.1126/scitranslmed.adk3259 PMC12336440

[12] S. B. Halstead, S. Rojanasuphot, N. Sangkawibha, Original antigenic sin in dengue. Am. J. Trop. Med. Hyg. **32**, 154–156 (1983). 6824120 10.4269/ajtmh.1983.32.154

[13] H. Clapham , Dengue virus (DENV) neutralizing antibody kinetics in children after symptomatic primary and postprimary DENV infection. J. Infect. Dis. **213**, 1428–1435 (2016). 26704615 10.1093/infdis/jiv759 PMC4813744

[14] M. T. Alera , Incidence of Dengue virus infection in adults and children in a prospective longitudinal cohort in the Philippines. PLoS Negl. Trop. Dis. **10**, e0004337 (2016). 26845762 10.1371/journal.pntd.0004337 PMC4742283

[15] H. Salje , Evaluation of the extended efficacy of the Dengvaxia vaccine against symptomatic and subclinical dengue infection. Nat. Med. **27**, 1395–1400 (2021). 34168334 10.1038/s41591-021-01392-9 PMC8364868

[16] M. Hamins-Puértolas , Household immunity and individual risk of infection with dengue virus in a prospective, longitudinal cohort study. Nat. Microbiol. **9**, 274–283 (2023). 38110699 10.1038/s41564-023-01543-3 PMC10895643

[17] L.C. Katzelnick , Antigenic evolution of dengue viruses over 20 years. Science **374**, 999–1004 (2021). 34793238 10.1126/science.abk0058 PMC8693836

[18] R. V. Gibbons , Analysis of repeat hospital admissions for dengue to estimate the frequency of third or fourth dengue infections resulting in admissions and dengue hemorrhagic fever, and serotype sequences. Am. J. Trop. Med. Hyg. **77**, 910–913 (2007). 17984352

[19] A. Quirine , Contributions from the silent majority dominate dengue virus transmission. PLoS Pathog. **14**, e1006965 (2018). 29723307 10.1371/journal.ppat.1006965 PMC5933708

[20] V. Duong , Asymptomatic humans transmit dengue virus to mosquitoes. Proc. Natl. Acad. Sci. U.S.A. **112**, 14688–14693 (2015). 26553981 10.1073/pnas.1508114112 PMC4664300

[21] A. T. Huang , Assessing the role of multiple mechanisms increasing the age of dengue cases in Thailand. Proc. Natl. Acad. Sci. U.S.A. **119**, e2115790119 (2022). 35533273 10.1073/pnas.2115790119 PMC9171776

[22] A. Utarini , Efficacy of Wolbachia-infected mosquito deployments for the control of dengue. N. Engl. J. Med. **384**, 2177–2186 (2021). 34107180 10.1056/NEJMoa2030243 PMC8103655

[23] H. Salje, Achieving zero deaths from dengue virus under evolving population immunity. Lancet Infect. Dis. **24**, 12–13 (2023). 37979587 10.1016/S1473-3099(23)00691-6

[24] D. Weiskopf , Dengue virus infection elicits highly polarized CX3CR1+ cytotoxic CD4+ T cells associated with protective immunity. Proc. Natl. Acad. Sci. U.S.A. **112**, E4256–E4263 (2015). 26195744 10.1073/pnas.1505956112 PMC4534238

[25] L. Yang, B. T. Grenfell, M. J. Mina, Waning immunity and re-emergence of measles and mumps in the vaccine era. Curr. Opin. Virol. **40**, 48–54 (2020). 32634672 10.1016/j.coviro.2020.05.009

[26] P. Chhabra , Homotypic and heterotypic protection and risk of re-infection following natural norovirus infection in a highly endemic setting. Clin. Infect. Dis. **72**, 222–229 (2020). 10.1093/cid/ciaa019 PMC7840104 33501947

[27] S. Wraith , Homotypic protection against influenza in a pediatric cohort in Managua, Nicaragua. Nat. Commun. **13**, 1190 (2022). 35246548 10.1038/s41467-022-28858-9 PMC8897407

[28] A. L. Rothman, Immunity to dengue virus: A tale of original antigenic sin and tropical cytokine storms. Nat. Rev. Immunol. **11**, 532–543 (2011). 21760609 10.1038/nri3014

[29] J. Andrade, Code and data for DENV reinfection analysis. GitHub. https://github.com/jandraor/DENV_reinfections. Accessed 10 May 2026.

