# Supplementary material for: Long-term antibody dynamics challenge the paradigm of lifelong homotypic immunity to dengue virus
Source: Proc Natl Acad Sci U S A. 2026 May 27;123(22):e2606206123. doi: 10.1073/pnas.2606206123 (PMC13229109; doi:10.1073/pnas.2606206123)
Supplement: Supplementary file 1 — Appendix 01 (PDF) [file pnas.2606206123.sapp.pdf]

Supplementary

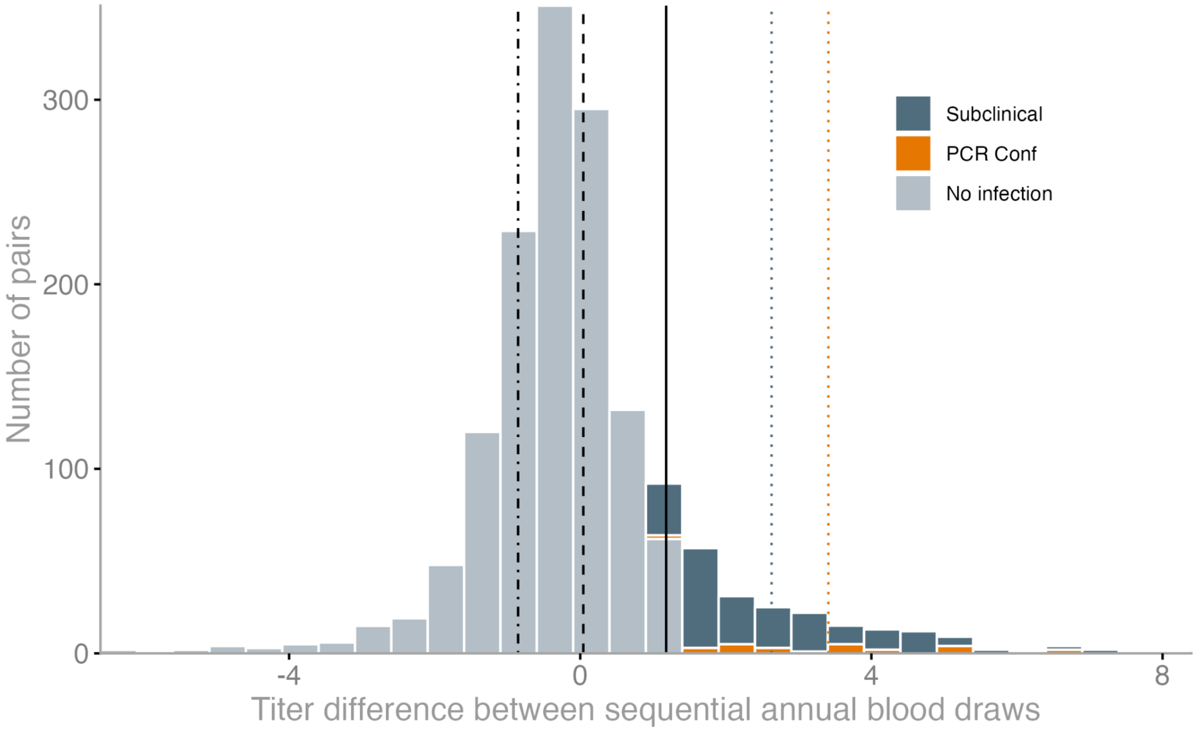

Figure S1. Detection of subclinical infections. Histogram of differences in antibody titers between sequential annual blood draws in the NMC cohort. Pairs were classified as no infection (grey), PCR-confirmed infection (orange), or subclinical infection (blue). The solid vertical line indicates the predefined serological threshold for infection (titer difference ≥ 1.18). The dashed vertical line shows the overall mean titer difference across all annual pairs, while the dot–dash line indicates the mean titre change among pairs showing antibody decay (titer difference < 0). Dotted vertical lines denote the mean titre differences for PCR-confirmed infections (orange) and subclinical infections (blue).

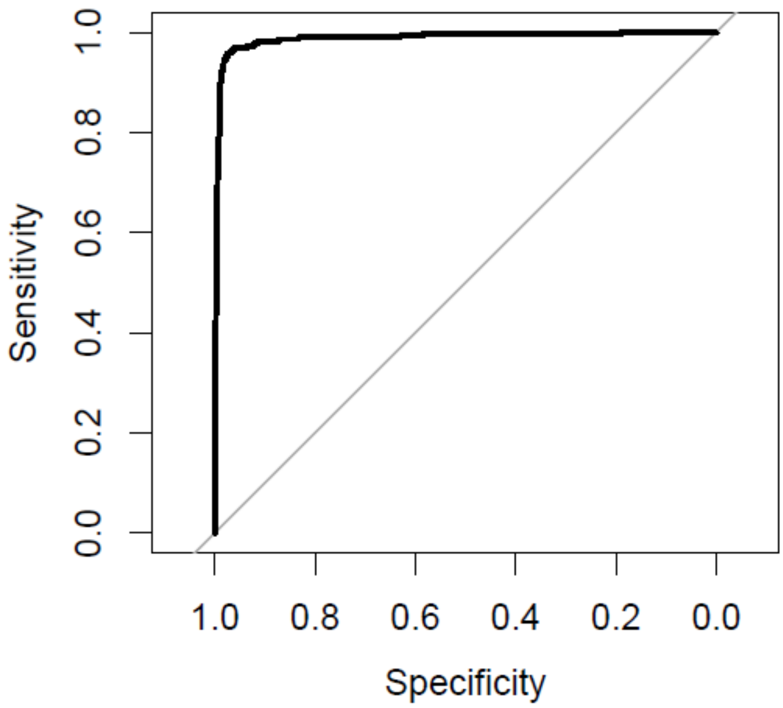

Figure S2: ROC curve to determine the infection threshold in the NMC cohort. The selected threshold of 1.18 yielded an AUC of 0.99.

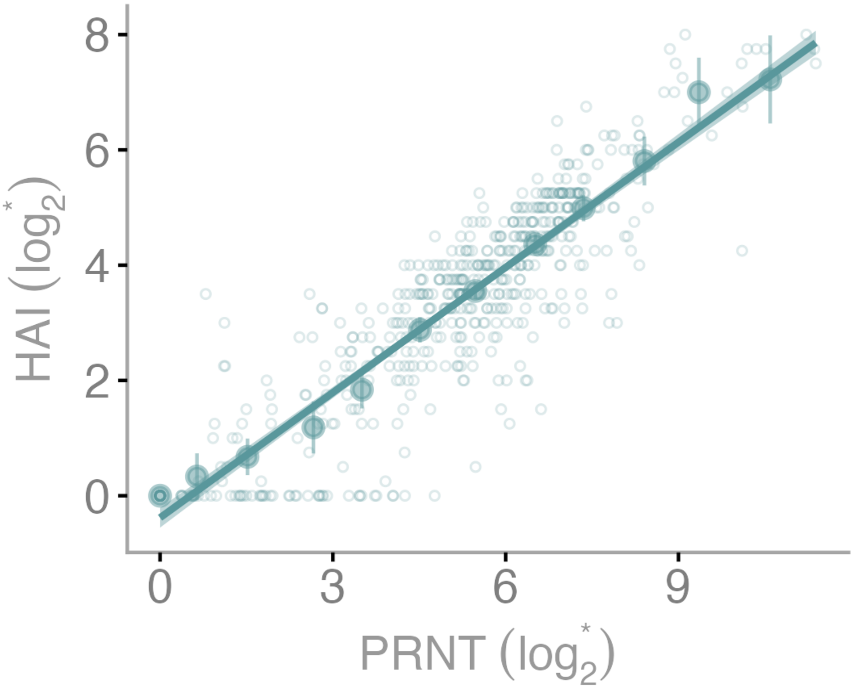

Figure S3: Correlation between HAI and PRNT. Among 560 blood samples from 112 individuals with both assays available, the Pearson correlation coefficient was 0.89.

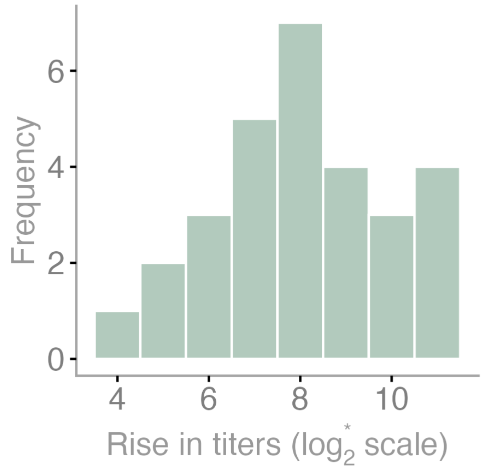

Figure S4. Mean rise in log2-titers following PCR confirmed symptomatic infection. Histogram of the difference in log2-titers measured in the blood draw prior to a PCR-confirmed symptomatic infection event and the log2-titer measured in the acute blood draw in the NMC cohort.

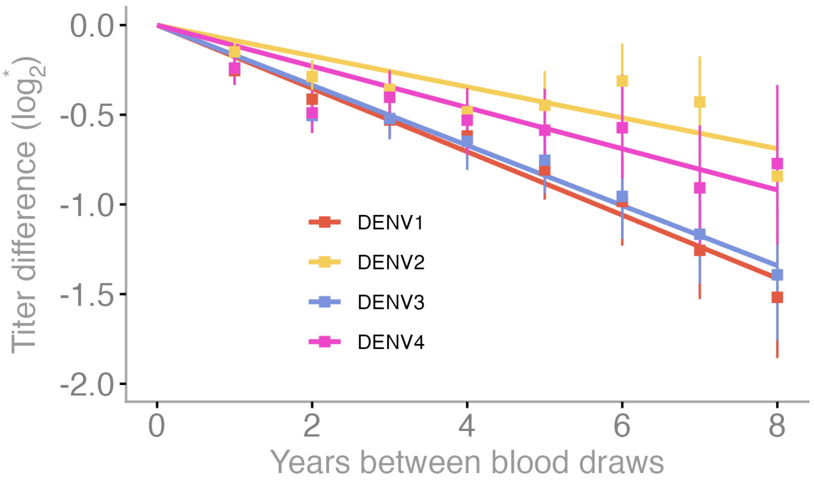

Figure S5. Serotypic NMC long-term decay rates. Observed difference in log-titers (PRNT) by serotype. The solid lines represent the fit from a separate linear model to each serotype.

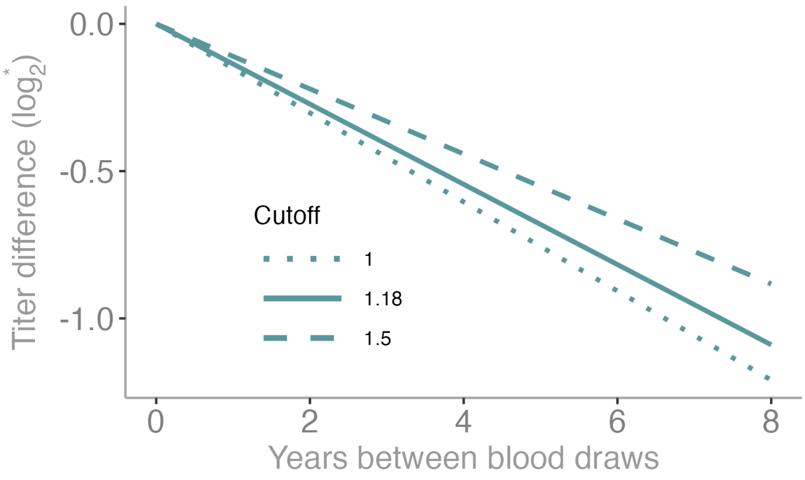

Figure S6. Sensitivity of the estimated long-term decay rate to the detection threshold for subclinical infections in the NMC cohort. The average decay rate was estimated from changes in log titers between any two blood draws from the same individual and infection event. The first blood draw of each infection event was excluded to avoid confounding effects from short-term dynamics. The dotted, solid and dashed lines correspond to thresholds of 1, 1.18 and 1.5, respectively. The corresponding long-term decay rates are 0.15 [0.13 - 0.17], 0.14 [0.12 - 0.15] and 0.11 y^{-1} [0.10 - 0.12].

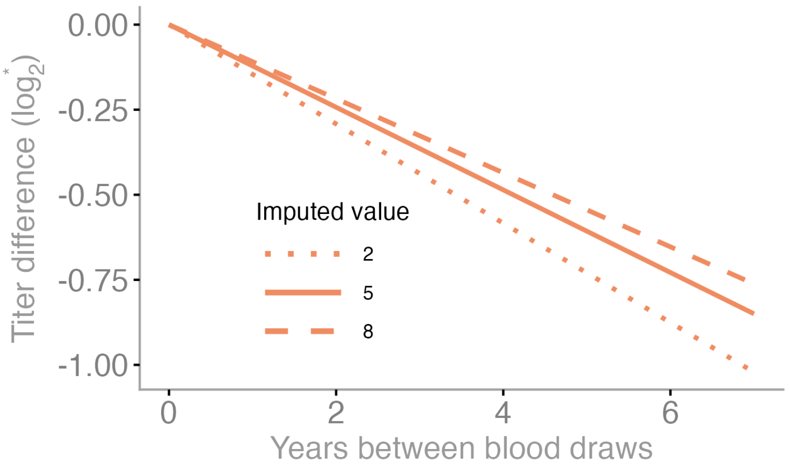

Figure S7. Sensitivity of the estimated long-term decay rate to the value imputed to measurements below the limit of detection in the KFCS cohort. The average decay rate was estimated from changes in log titers between any two blood draws from the same individual and infection event. The first blood draw of each infection event was excluded to avoid confounding effects from short-term dynamics. The dotted, solid and dashed lines correspond to imputed values of 2, 5 and 8, respectively. The corresponding long-term decay rates are 0.15 [0.12 - 0.18], 0.12 [0.10 - 0.14] and 0.11 y^{-1} [0.09 - 0.13].

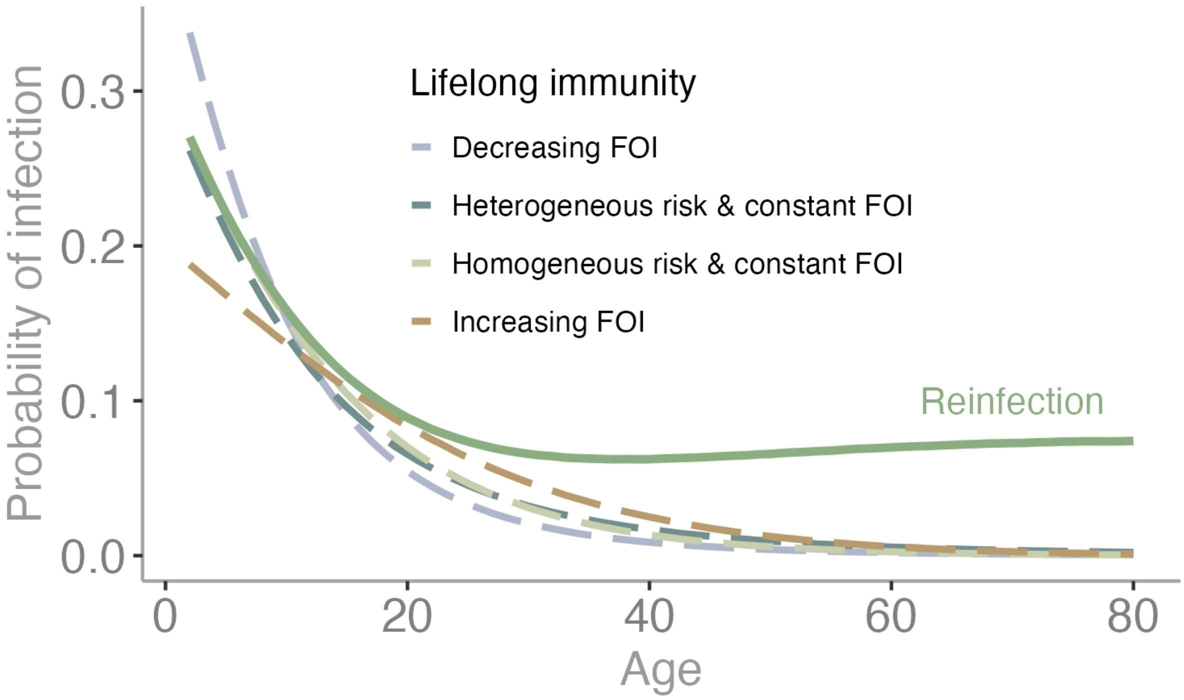

Figure S8. Age-specific probability of infection under alternative force-of-infection (FOI) assumptions in the CPC cohort. Dashed lines show the expected age-specific probability of infection for four homotypic lifelong immunity scenarios: decreasing FOI, increasing FOI, homogeneous individual risk with constant FOI, and heterogeneous individual risk with constant FOI. The solid line represents the constant-FOI reinfection scenario.

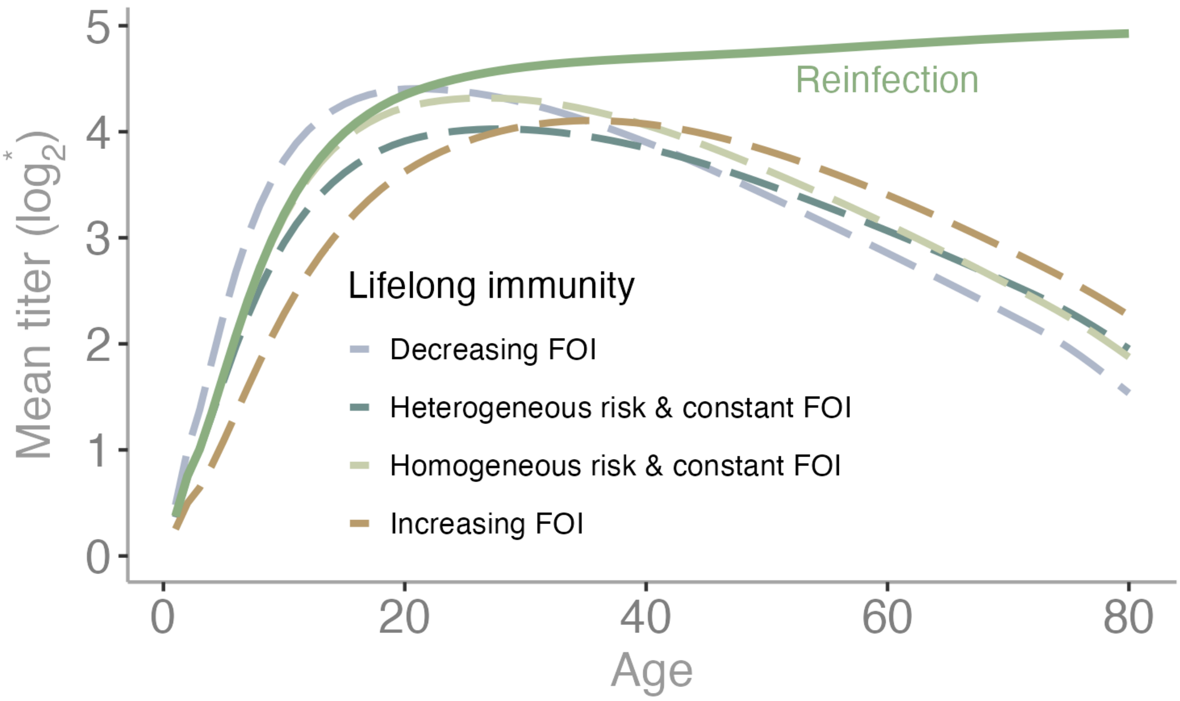

Figure S9. Population-level mean DENV antibody titer patterns by age under alternative force-of-infection (FOI) assumptions in the CPC cohort. Dashed lines show the expected mean titer by age for four homotypic lifelong immunity scenarios: decreasing FOI, increasing FOI, homogeneous individual risk with constant FOI, and heterogeneous individual risk with constant FOI. The solid line represents the constant-FOI reinfection scenario.

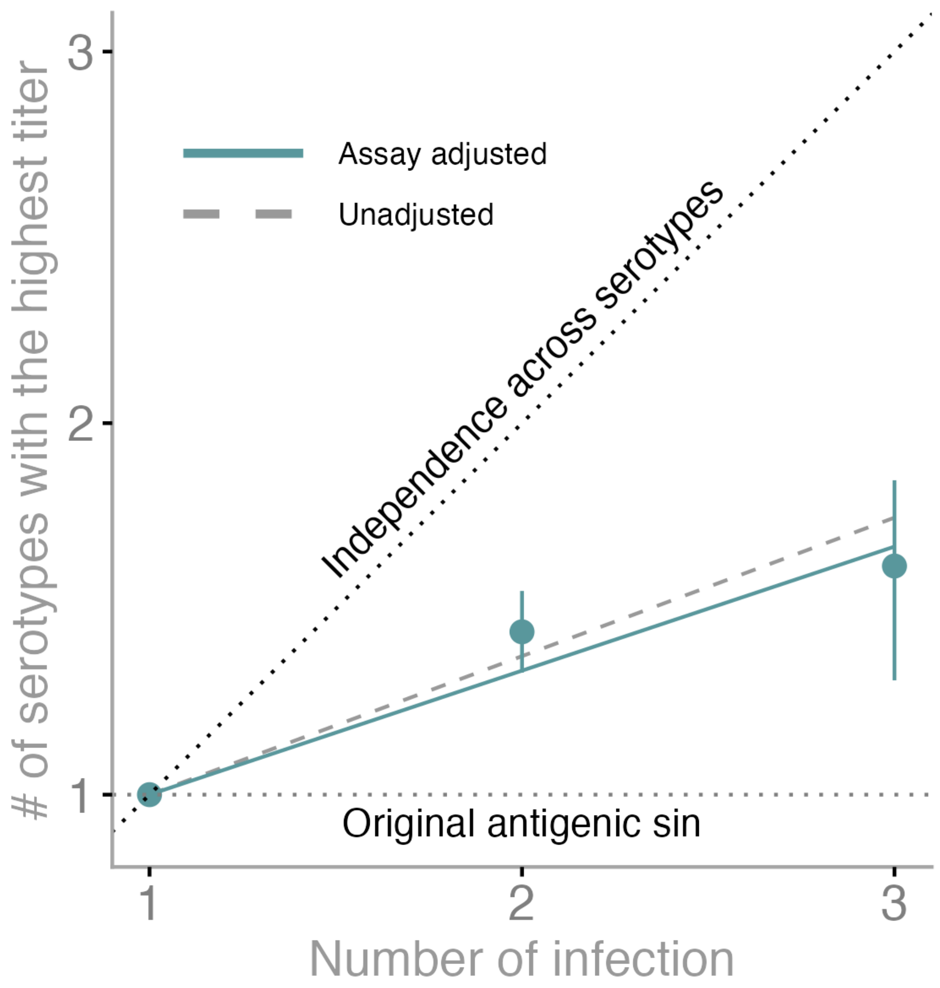

Figure S10. Imprinting of infections in the NMC cohort. Among individuals with one, two, or three infections, we show the number of unique serotypes exhibiting the highest antibody titers in the subsequent annual blood draw following infection. The dotted lines represent the expected patterns if serotype-specific responses were dominated by a single serotype (horizontal line, consistent with original antigenic sin) or if all serotypes were independent, such that only the serotype of the most recent infection determines the highest titer. The solid line, dots, and vertical bars represent the results adjusted for serotype-specific titers. To account for some serotypes always generating higher titers due to the antigenic properties of the virus in the assay, we centred each titer around the mean titer for that serotype. The dashed line represents the analysis using crude titers.

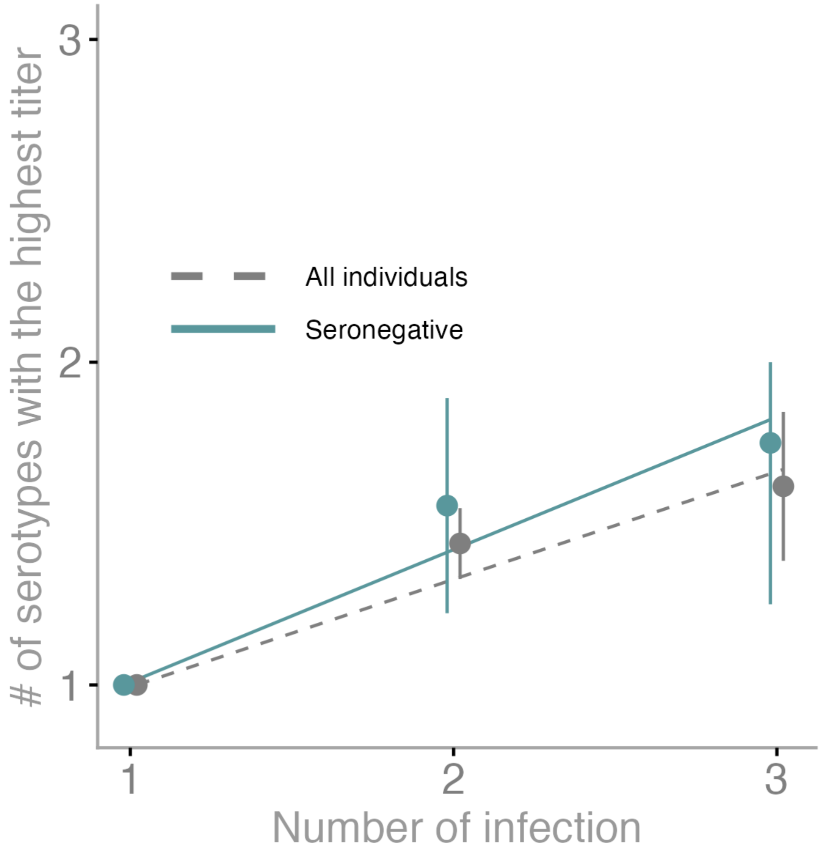

Figure S11. **Imprinting of infections in seronegative individuals in the NMC cohort**. Among individuals with one, two, or three infections, we show the number of unique serotypes exhibiting the highest antibody titers in the subsequent annual blood draw following infection. The grey dashed line, points, and vertical bars represent results adjusted for serotype-specific titers across all individuals, regardless of serostatus. To account for differences in baseline titer levels across serotypes due to antigenic properties in the assay, each titer was centred by subtracting the mean titer for that serotype. The teal solid line, points, and vertical bars represent results for seronegative individuals.

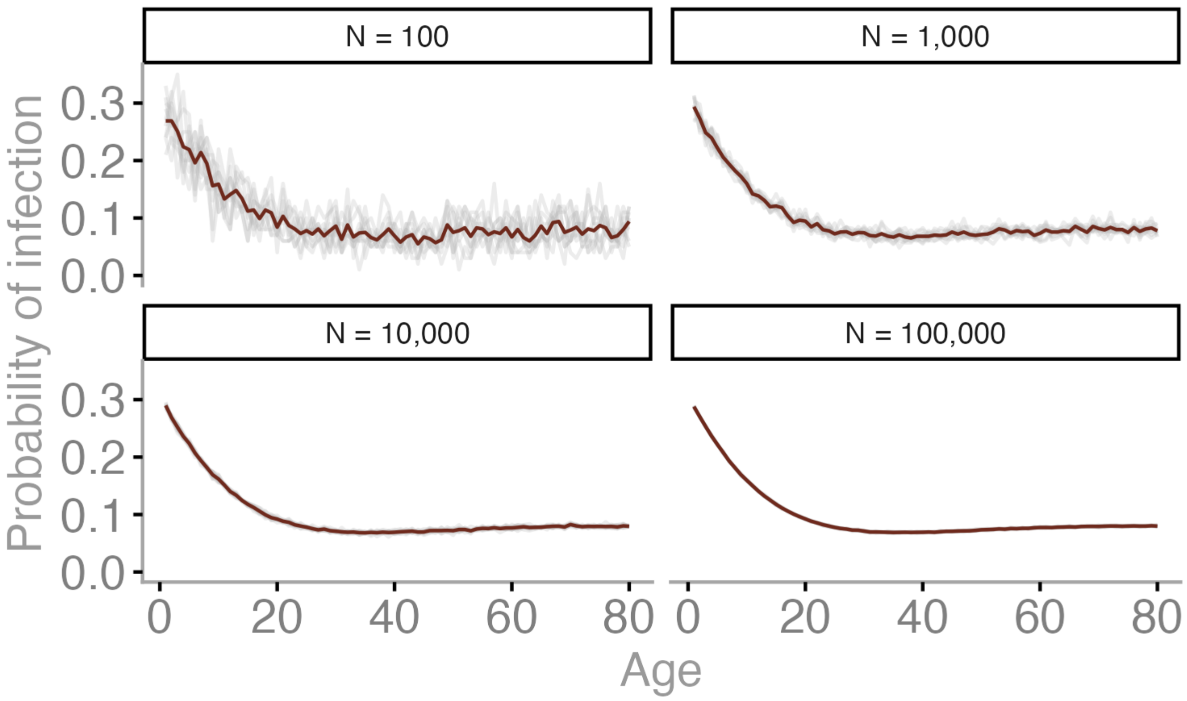

Figure S12. Probability of infection under various population sizes. Each panel contains 10 simulations (grey lines) of the catalytic model for a given force of infection (0.085), rate of loss of immunity (0.0075), and population size (N). The brown line represents the mean.

Table S1. Parameter estimates from the model fitted to population age-specific titers and infections

Parameter	Definition	Estimate (95% CI)
λ_{CPC}	Annual per-serotype probability of infection among individuals in the CPC cohort.	0.085 (0.080-0.089)
λ_{KFCS}	Annual per-serotype probability of infection among individuals in the KFCS cohort.	0.054 (0.053-0.055)
ρ	Annual loss of immunity to homotypic reinfection.	0.0064 (0.0059-0.0076)
$log^*{}_2A^1{}_0$	Starting titer following a primary infection in the adjusted $\log_2$ scale.	1.30 (1.29-1.57)
ϕ	Asymptotic upper bound of antibody titers.	5.64 (5.51-5.70)
σ	Standard deviation of antibody titers in the adjusted $\log_2$ scale.	2.66 (2.53-2.88)

Table S2. Maximum likelihood estimates from the model fitted to population age-specific titers and infections, assuming no homotypic reinfection ($\rho = 0$).

Parameter	Estimate (95% CI)
λ_{CPC}	0.0565 (0.0540-0.0593)
λ_{KFCS}	0.0389 (0.0380-0.0391)
$log^*{}_2A^1{}_0$	1.87 (1.74-1.87)
ϕ	6.88 (6.86-6.93)
σ	2.47 (2.32-2.69)
